# Fungicides With Contrasting Mode of Action Differentially Affect Hyphal Healing Mechanism in *Gigaspora* sp. and *Rhizophagus irregularis*

**DOI:** 10.3389/fpls.2021.642094

**Published:** 2021-03-10

**Authors:** Victor Hugo Rodriguez-Morelos, Maryline Calonne-Salmon, Vincent Bremhorst, Mónica Garcés-Ruiz, Stéphane Declerck

**Affiliations:** ^1^Laboratory of Mycology, Earth and Life Institute, Université catholique de Louvain, Louvain-la-Neuve, Belgium; ^2^Louvain Institute of Data Analysis and Modeling in Economics and Statistics, Statistical Methodology and Computing Service, Université catholique de Louvain, Louvain-la-Neuve, Belgium

**Keywords:** arbuscular mycorrhizal fungi, extraradical mycelium, hyphal healing mechanism, fungicides, growing hyphal tips

## Abstract

Fungicides are widely used in conventional agriculture to control fungal diseases, but may also affect non-target microorganisms such as arbuscular mycorrhizal (AM) fungi. These root symbionts develop extended mycelial networks within the soil via mechanisms such as anastomosis that indistinctly concerns intact and damaged hyphae, the latter being named hyphal healing mechanism (HHM). The HHM differs between *Glomeraceae* and *Gigasporaceae*. However, the effects of fungicides on this mechanism in unknown. Here, the impact of azoxystrobin, pencycuron, flutolanil, and fenpropimorph at 0.02 and 2 mg L^–1^ were tested *in vitro* on the HHM of *Gigaspora* sp. MUCL 52331 and *Rhizophagus irregularis* MUCL 41833, and repair events visualized carefully under a dissecting bright-field light microscope. Azoxystrobin was the more detrimental for both AM fungi at 2 mg L^–1^, while fenpropimorph impacted only *R. irregularis* (stimulating at low and inhibiting at high concentration). Conversely, flutolanil and pencycuron did not impact any of the two AM fungi. The mechanisms involved remains to be elucidated, but perturbation in the still-to-be firmly demonstrated spitzenkörper or in sterols content as well as a process of hormesis are possible avenues that deserve to be explored in view of a rationale management of chemicals to control fungal pathogens without harming the beneficial AM fungi.

## Introduction

The extraradical mycelium (ERM) of arbuscular mycorrhizal (AM) fungi is fundamental in plant nutrition and maintenance of biological fertility in agroecosystems (Pepe et al., 2018; de Novais et al., 2019a). Keeping the integrity of this belowground network is thus essential, not only for the survival of these fungi but also for their manifold benefits to plants. Mechanisms such as anastomosis and healing are pivotal for the spread and maintenance/survival of AM fungal colonies (de la Providencia et al., 2005). Both mechanisms drastically differ between genera (de la Providencia et al., 2005; Voets et al., 2006) and could be affected by agricultural practices such as plowing (Brito et al., 2012) or application of pesticides (de Novais et al., 2019b; Hage-Ahmed et al., 2019).

Anastomosis, that is the process of fusion between branches of the same or different hyphae to constitute a mycelial network (Kirk et al., 2008), has been abundantly reported in the fungal kingdom (Glass et al., 2000) and described in intact two-dimensional (Giovannetti et al., 2004) as well as three-dimensional (de la Providencia et al., 2005) ERM networks of AM fungi. It indistinctly concern intact and damaged hyphae, the latter being described as a hyphal healing mechanism (HHM) (de la Providencia et al., 2007). The HHM has been described in four successive events. First, it begins with the formation of a septum near or in the apical zone on both sides of the injured hyphae, which prevents massive cytoplasmic/protoplasmic leakage into the surrounding environment. Secondly, one or several growing hyphal tips (GHTs) emerge from both extremities of the cut hyphae either protruding through the septum or emerging behind it. Thirdly, the GHTs elongate and grow toward each other and in most cases enter into contact. Fourthly, fusion is observed between GHTs with re-establishment of cytoplasmic/protoplasmic flow (de la Providencia et al., 2005). Importantly, the HHM differ between *Glomeraceae* and *Gigasporaceae*. Indeed, the HHM in *Glomeraceae* is oriented toward the reconnection of the affected area by linking several hyphae in relatively small vicinity or by recolonization of roots and substrate, by contrast to *Gigasporaceae* in which the HHM is nearly always oriented toward the re-establishment of hyphal integrity (de la Providencia et al., 2007). This suggests that both fungi have developed different strategies to grow and survive under adverse conditions (de la Providencia et al., 2005).

Fungicides are widely used in conventional agriculture to control fungal diseases. Unfortunately, these molecules may also affect soil plant-beneficial microorganisms such as AM fungi (Jin et al., 2013; Buysens et al., 2015). Their impact on these belowground microorganisms have been investigated under field (e.g., Schalamuk et al., 2014; Rivera-Becerril et al., 2017), greenhouse (e.g., Jin et al., 2013; Channabasava et al., 2015; Rabab and Reda, 2019) and growth chamber (de Novais et al., 2019a) conditions as well as *in vitro* on root organs (e.g., Campagnac et al., 2008; Zocco et al., 2008) or whole plants (e.g., Zocco et al., 2011).

*In vitro* cultivation systems offer several advantages, among which, the absence of any confounding effects caused by unwanted contaminants or environmental factors (e.g., soil physico-chemical parameters) and the easy non-destructive observations of growth and development of fungi. Spore germination (Chiocchio et al., 2000; Zocco et al., 2008; Buysens et al., 2015), root colonization (Campagnac et al., 2008, 2009; Calonne et al., 2010, 2012), anastomosis formation (Cardenas-Flores et al., 2011; de Novais et al., 2019a,b), sterol biosynthesis pathway (Campagnac et al., 2010; Calonne et al., 2012) and transport of nutrients (e.g., phosphorus) from fungus to plant (Zocco et al., 2011) have been investigated *in vitro* in presence of different types of fungicides. Results differed significantly with fungicide and dose of application. However, no study has reported the effects of fungicides on the HHM and thus on the ability of hyphae to maintain integrity following physical disturbance.

Azoxystrobin, pencycuron, flutolanil and fenpropimorph are amongst the most frequently used fungicides to control soil fungal diseases. Azoxystrobin is a systemic fungicide that belongs to the class of methoxyacrylates, which are derived from the naturally occurring strobilurins. It is the most widely sold fungicide worldwide (Zhang Q. et al., 2019), used against several fungal diseases of many edible crops and ornamental plants. It exhibits its fungicidal activity by binding to the quinol oxidation (Q_o_) site of cytochrome b to inhibit mitochondrial respiration in fungal species from the Ascomycota, Basidiomycota, and Deuteromycota and fungal-like species from the Oomycota (Bartlett et al., 2002; Feng et al., 2020). Pencycuron is a phenylurea fungicide of contact, which is highly specific to *Rhizoctonia solani*, inhibiting mycelial growth by blocking cell division and destroying the cytoskeleton of the microtubules during mitosis (Young, 2012). Flutolanil is a systemic phenyl benzamide fungicide, used against diseases caused by Basidiomycota in crop plants (Zhao et al., 2019). It mainly inhibits the hyphal growth and infection formation, and strongly reduce the mycelial O_2_ consumption of *R. solani* as well as the activity of succinate dehydrogenase complex (Complex II) in mitochondria (Mol et al., 2019). Finally, fenpropimorph is a morpholine of broad-spectrum considered as a sterol biosynthesis inhibitor (SBI). It specifically inhibits at low concentrations the sterol Δ^8^→Δ^7^-sterol isomerase, and additionally, when it is used at higher concentrations, inhibits Δ^14^-sterol reductase (Marcireau et al., 1990). This fungicide is mainly applied in cereals to control *Blumeria* (powdery mildew) and *Puccinia* (cereal rust) species (Stenzel and Vors, 2019).

A number of studies under strict *in vitro* culture conditions have investigated the effects of these fungicides on AM fungi. Azoxystrobin and its formulation Amistar did not impact spore germination and root colonization of potato associated with *R. irregularis* MUCL 41833 at threshold concentration (IC50 ≤ 0.1 mg L^–1^ a.i.) for the control of *R. solani*, while at 10 times this threshold, spores production and mycelium development were significantly affected (Buysens et al., 2015). In the same study, at threshold value for the control of *R. solani*, pencycuron and its formulation Monceren, did not affect spore germination and intra- or ERM development (Buysens et al., 2015). Finally, flutolanil and its formulation Monarch at threshold value for the control of *R. solani* did not affect spores germination or ERM development but decreased root colonization and arbuscules formation. In other studies, using root organ cultures (ROC) (Campagnac et al., 2008, 2009, 2010; Zocco et al., 2008; Oger et al., 2009) or whole plants (Zocco et al., 2011), the effects of fenpropimorph on the AM fungal symbiosis was demonstrated. Fenpropimorph presented a high toxicity with drastic sterols modifications in the host roots which was mirrored by a drastic reduction of root growth, root colonization and decrease of phosphorus transport, alkaline phosphatase and succinate dehydrogenase activities of the ERM (Zocco et al., 2011). All these studies suggested that fungicides may have undesirable effects on AM fungi, but none considered their effects on HHM.

The objective of this study was to investigate under *in vitro* culture conditions the impact of two different concentrations (0.02 and 2 mg L^–1^) of azoxystrobin, pencycuron, flutolanil, and fenpropimorph on the HHM of two AM fungi (*Gigaspora* sp. MUCL 52331 and *R. irregularis* MUCL 41833) belonging to phylogenetically distant families and thus having different life history strategies.

## Materials and Methods

### Biological Material

The AM fungi *Gigaspora* sp. (Gerdemann and Trappe) MUCL 52331 and *Rhizophagus irregularis* (Błaszk., Wubet, Renker, and Buscot) C. Walker and A. Schüßler as [“irregulare”] MUCL 41833 were supplied by the Glomeromycota *in vitro* collection (GINCO –^1^). Both strains were maintained in association with Ri T-DNA transformed chicory (*Cichorium intybus* L.) roots on 135 mm diam. Petri plates containing 100 ml Modified Strullu–Romand (MSR) medium (Declerck et al., 1998).

### Fungicide Medium Preparation

The active ingredients (a.i.) of four fungicides that disrupt respiration (azoxystrobin and flutolanil), cytoskeleton and motor proteins (pencycuron) and sterol biosynthesis in membranes (fenpropimorph) (Fungicide Resistance Action Committee, 2020) were supplied by Sigma-Aldrich, Inc., [Darmstadt, Germany]. Each fungicide was dissolved in a solution of acetone (5 ml L^–1^ of MSR medium) and added to a bottle containing 50 ml sterilized (121°C for 15 min) MSR medium. The a.i. were added at a concentration of 0.02 and 2 mg L^–1^ MSR medium (Zocco et al., 2008). The bottles contained a stirring magnet to avoid solidification and obtain a homogeneous concentration of the different a.i. One bottle containing MSR medium without fungicides but added with acetone (MSR^*acetone*^) and another with MSR medium alone (MSR^*control*^) were used as controls.

### Experimental Design

**FIGURE 1:**
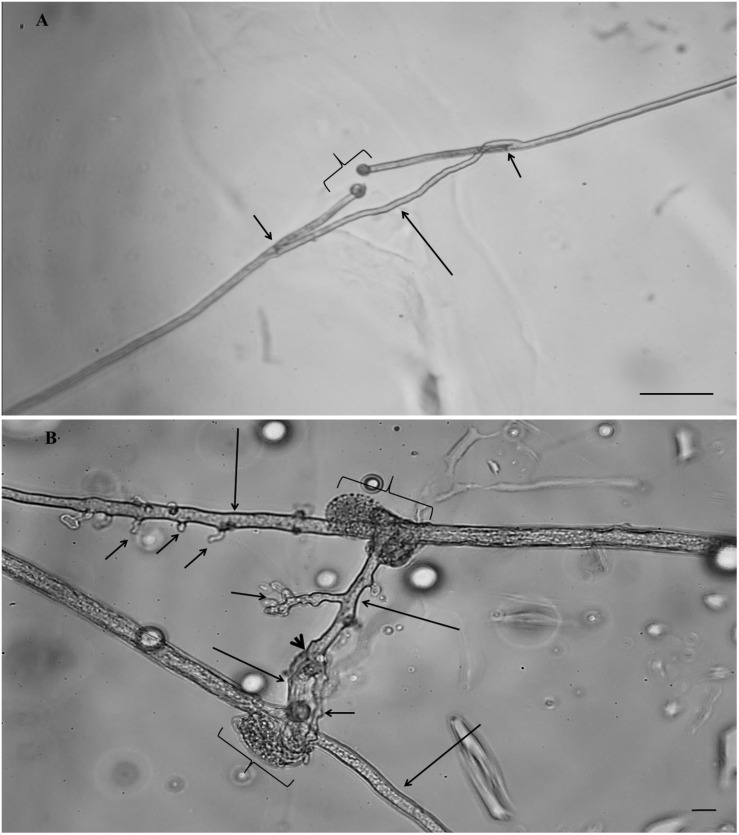
Hyphal healing mechanism (HHM) of AM fungi in the control treatment **(A)** *Gigaspora* sp. MUCL 52331, the plugs were formed at both extremities in front of the hyphal injury (braces), two growing hyphal tips (GHTs) emerged at both extremities behind the septum (short arrows) and fused (large arrow) with re-establishment of cytoplasmic/protoplasmic flow (scale bar 50 μm). **(B)** *Rhizophagus irregularis* MUCL 41833, the plugs were formed at both extremities in front of the hyphal injury (braces), two GHTs from each injury site emerged through the septum (large arrows) and one GHT of each side contacted and fused (arrowhead) with re-establishment of cytoplasmic/protoplasmic flow. Several hyphal branches (short arrows) developed from the GHTs (scale bar 10 μm).

The Petri plates were placed in a slope (∼10°) during filling of the MSR medium so that medium was thinner at one side of the Petri plate at solidification. One chicory root piece was placed in the thicker side of the Petri plate and associated with one of the AM fungi. For *Gigaspora* sp., a 3 cm × 3 cm piece of gel containing mycelium and three spores from a 6-month-old ROC was placed close to the root, while for *R. irregularis*, a 2.5 cm × 2.5 cm sliced piece of gel containing mycelium and approximately 500 spores from a 2-month-old ROC was used. The Petri plates were incubated in an inverted position at 27°C in a growth chamber under dark conditions. After 12 and 6 weeks of growth for *Gigaspora* sp. and *R*. *irregularis*, respectively, Petri plates showing adequate ERM development were selected. Between 6 and 10 hyphae growing on the surface of the MSR medium in the thinner part of the Petri plates and showing dense cytoplasmic/protoplasmic flow under a dissecting bright-field light microscope (Olympus SZ2-CTV, Japan) at 40× magnification were selected in each Petri plate. The selected hyphae were chosen at a reasonable distance from each other to avoid as much as possible interferences (i.e., hyphae contact from different cuts). Under horizontal laminar hood, hyphae were cut with a sterilized scalpel under the dissecting bright-field light microscope at 6.7× to 40× magnification. Immediately after cutting, 20 μl of MSR medium without fungicides (MSR^control^) or containing one of the two concentrations of fungicides or containing only acetone (MSR^acetone^) was immediately added at the place of injury. The HHM was analyzed in injured hyphae belonging to two AM fungal colonies (i.e., two Petri plates) per strain (*Gigaspora* sp. and *R. irregularis*) and for each control treatment (MSR^treatment^, MSR^Acetone^) and fungicide concentration (0.02 and 2 mg L^–1^). The fungicide treatments were labeled as follows: azoxystrobin^0.02 mgL^–1^^, azoxystrobin^2 mgL^–1^^, flutolanil^0.02 mgL^–1^^, flutolanil^2 mgL^–1^^, pencycuron^0.02 mgL^–1^^, pencycuron^2 mgL^–1^^, fenpropimorph^0.02 mgL^–1^^, and fenpropimorph^2 mgL^–1^^. The cut hyphae were observed cautiously at regular intervals under the dissecting bright-field light microscope at 100× or 200× magnification, first frequently during the first 60 min, then each hour until 16 h and finally after 24, 30, 36, and 48 h. The hyphae were visualized carefully under a dissecting bright-field light microscope (Olympus BH2–RFCA, Japan) at 40× or 100× magnification (Figures 1A,B).

### Data Collection

**FIGURE 2:**
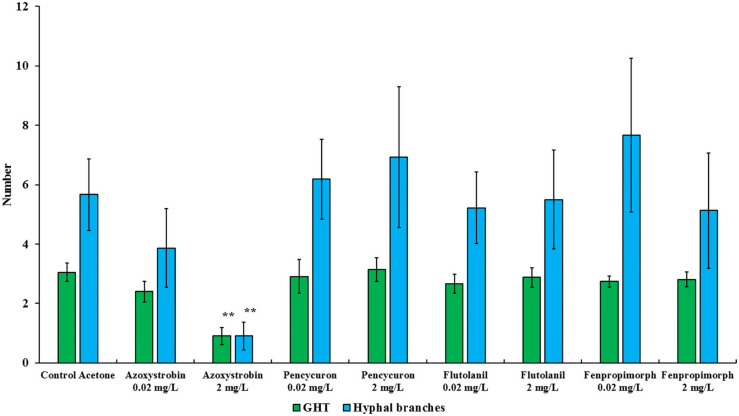
Number of growing hyphal tips (GHTs) and their hyphal branches produced in the hyphal healing mechanism (HHM) of *Gigaspora* sp. MUCL 52331 in absence (control acetone) or in presence of increasing concentrations of azoxystrobin, pencycuron, flutolanil and fenpropimorph (0.02 and 2 mg L^–1^). Data were obtained 48 h after hyphal physical injury and addition of MSR medium containing or not the fungicide at the place of hyphal injury. Kruskal-Wallis followed by a Dunn multiple comparison *post hoc* test was performed to validate significant difference between fungicides treatments and their respective control acetone: *, **, and *** indicate *P* < 0.05; 0.01 and 0.001, respectively. *P*-values below 0.05 are significant.

**FIGURE 3:**
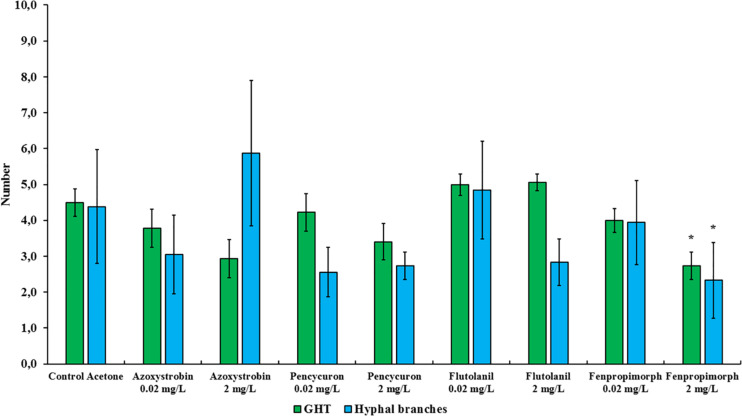
Number of growing hyphal tips (GHTs) and their hyphal branches produced in the hyphal healing mechanism (HHM) of *Rhizophagus irregularis* MUCL 41833 in absence (control acetone) or in presence of increasing concentrations of azoxystrobin, pencycuron, flutolanil and fenpropimorph (0.02 and 2 mg L^–1^). Data were obtained 48 h after hyphal physical injury and addition of MSR medium containing or not the fungicide at the place of hyphal injury. Kruskal-Wallis followed by a Dunn multiple comparison *post hoc* test was performed to validate significant difference between fungicides treatments and their respective control acetone: *, **, and *** indicate *P* < 0.05; 0.01 and 0.001, respectively. *P*-values below 0.05 are significant.

Following hyphae cutting, four events in the HHM (de la Providencia et al., 2005) were monitored: (1) septum formation near or in the apical zone on both sides of the cut hyphae (result not shown); (2) emission of GHTs through the septum or behind it and production of new branches on the GHTs; (3) elongation, orientation, and contact of the GHTs, and (4) fusion and re-establishment of cytoplasmic/protoplasmic flow. Additionally, the number of GHTs and total number of hyphal branches coming from the GHTs were counted at 48 h. The observed numbers are reported in Figures 2, 3. The percentage of observed events within each treatment and control group are reported in Table 1 and (Supplementary Table 2 for the MSR^control^ group).

**TABLE 1:** Descriptive data percentages of hyphal branches production, growing hyphal tips (GHTs) emission, contact and fusion of the hyphal healing mechanism (HHM) of *Gigaspora sp.* MUCL 52331 and *Rhizophagus irregularis* MUCL 41833 in absence (control acetone) or in presence of increasing concentrations of azoxystrobin, pencycuron, flutolanil and fenpropimorph (0.02 and 2 mg L^–1^).

	*Gigaspora* sp. MUCL 52331			*Rhizophagus irregularis* MUCL 41833		
		
	Control acetone	0.02 mg L^–1^	2 mg L^–1^	Control acetone	0.02 mg L^–1^	2 mg L^–1^
**Azoxystrobin**						
Hyphal branches (%)	94.4	86.7	35	73.7	66.7	50
GHT emission (%)	94.4	86.7	30	100	88.9	87.5
GHT contact (%)	88.9	86.7	15	73.7	66.7	37.5
GHT fusion (%)	88.9	73.3	10	68.4	55.5	31.2
Number of injured hyphae (n)	18	15	20	19	18	16
**Pencycuron**						
Hyphal branches (%)	81.2	81.8	64.3	94.4	88.9	86.7
GHT emission (%)	87.5	81.8	92.8	94.7	100	100
GHT contact (%)	81.2	81.8	85.7	89.5	83.3	73.3
GHT fusion (%)	81.2	81.8	71.4	73.7	72.2	60
Number of injured hyphae (n)	16	12	14	19	18	15
**Flutolanil**						
Hyphal branches (%)	72.2	83.3	81.2	94.7	100	100
GHT emission (%)	94.4	94.4	93.7	100	100	100
GHT contact (%)	83.3	77.7	93.7	89.5	90	88.9
GHT fusion (%)	83.3	72.2	75	73.7	75	77.8
Number of injured hyphae (n)	18	18	16	19	20	18
**Fenpropimorph**						
Hyphal branches (%)	87.5	86.7	56.2	85	86.7	70.6
GHT emission (%)	100	100	93.7	90	100	88.2
GHT contact (%)	100	93.3	87.5	85	80	35.3
GHT fusion (%)	87.5	73.3	75	70	73.3	29.4
Number of injured hyphae (n)	16	15	16	19	18	15

### Statistical Analysis

To evaluate the impact of the fungicides on emission, contact and fusion of GHTs, each hyphal injury from all treatments, was considered. A Cox proportional hazard model for right and interval censored (time-to-event) data was fitted using R software version 4.0.0. Specifically, the *MIICD.coxph* function of the *MIICD* R package was used (Delord, 2015). The non-observed events of the HHM after 48 h were considered as right censored. More information on the time-to-event statistical modeling framework and more precisely on the Cox model can be found in Cox (1972) and George et al. (2014). The hazard ratio’s (hr) reported in Table 2 and Supplementary Table 2 and defined as exp (β), where the β’s are the coefficient estimates obtained from the fit of the Cox model, are interpreted as follows: an hazard ratio greater (respectively, smaller) than one means that the event is more (respectively, less) likely [i.e., the event occurs more (respectively, less) often or is sooner (respectively, later) observed] within the given treatment group compared to the control. In our analysis, MSR^acetone^ is defined as the control group. For a seek of simplicity, Table 1 only reports the estimates (obtained from the Cox model) for the treatment groups. The ones corresponding to MSR^water^ are reported in Supplementary Table 1 since no significant difference was highlighted by the analysis between MSR^acetone^ and MSR^water^.

**TABLE 2:** Results of Cox proportional hazards regression analysis on growing hyphal tips (GHTs) emission, contact and fusion events of the hyphal healing mechanism (HHM) for *Gigaspora* sp. MUCL 52331 and *Rhizophagus irregularis* MUCL 41833 in presence of increasing concentrations of azoxystrobin, pencycuron, flutolanil and fenpropimorph (0.02 and 2 mg L^–1^).

		*Gigaspora* sp. MUCL 52331			*Rhizophagus irregularis* MUCL 41833		
			
	Fungicide concentration (mg L^–1^)	β	Hazard ratio	*P*-value	β	Hazard ratio	*P*-value
**Azoxystrobin**							
GHT emission	0.02	−0.392	0.676	0.296	−0.457	0.633	0.187
	2	−2.009	0.134	**<0.001**	−0.685	0.504	0.056
GHT contact	0.02	−0.424	0.655	0.259	−0.200	0.818	0.613
	2	−2.444	0.064	**<0.001**	−1.147	0.317	**0.020**
GHT fusion	0.02	−0.568	0.567	0.140	−0.345	0.708	0.416
	2	−3.129	0.044	**<0.001**	−1.224	0.294	**0.021**
**Pencycuron**							
GHT emission	0.02	0.083	1.086	0.847	0.484	1.623	0.164
	2	0.124	1.133	0.748	0.520	1.682	0.154
GHT contact	0.02	0.243	1.275	0.578	0.114	1.120	0.749
	2	−0.050	0.951	0.901	−0.135	0.873	0.728
GHT fusion	0.02	−0.092	0.912	0.840	0.258	1.295	0.506
	2	−0.311	0.733	0.464	−0.151	0.860	0.725
**Flutolanil**							
GHT emission	0.02	0.628	1.873	0.073	NA	NA	NA
	2	0.403	1.496	0.267	NA	NA	NA
GHT contact	0.02	0.178	1.195	0.633	0.075	1.078	0.825
	2	0.539	1.714	0.144	0.002	1.002	0.995
GHT fusion	0.02	0.049	1.050	0.899	0.139	1.149	0.709
	2	−0.222	0.801	0.569	0.120	1.127	0.752
**Fenpropimorph**							
GHT emission	0.02	0.139	1.149	0.711	1.275	3.580	**<0.001**
	2	−0.415	0.661	0.282	−0.345	0.708	0.334
GHT contact	0.02	−0.475	0.622	0.201	0.805	2.238	**0.033**
	2	−0.414	0.661	0.263	−1.294	0.274	**0.007**
GHT fusion	0.02	−0.538	0.584	0.185	0.638	1.893	0.109
	2	−0.247	0.781	0.533	−1.224	0.294	**0.019**

*Values were considered significant at *P* < 0.05 compared to the acetone control treatment. β = Regression coefficient. Hazard ratio = Exp (β). NA, Not applicable.*

In addition, the number of GHTs and hyphal branches produced were subjected to Kruskal–Wallis non-parametric test followed by a *post hoc* Dunn multiple comparison test to validate significant difference between fungicides treatments and their respective control acetone using the software IBM SPSS statistic 26 software. For all analyses, statistical significance was established at a 95% confidence level (i.e., when the *p*-value is less than 0.05).

## Results

### Hyphal Healing Mechanism in *Gigaspora sp.* and *R. irregularis* in the MSR^acetone^ and MSR^control^ Treatments

Whatever the AM fungus, a loss of cytoplasmic/protoplasmic content was observed at both extremities of the cut hyphae (Figures 1A,B). A septum was formed and a change in color inside the hyphae (dark brown to brown) was noticed within the first 180 min. The mechanism of HHM slightly differed between both AM fungi, especially in the number of GHTs produced. With *Gigaspora* sp., a low number of GHTs (with rare hyphal branches) was noticed emerging behind both extremities of the injured hyphae (Figure 1A). In most of the cases the GHTs grew toward each other, reconnected and re-established cytoplasmic/protoplasmic flow. In contrast, *R*. *irregularis* produced a high number of GHTs at both extremities growing through the septum and often bearing several hyphal branches. Hyphal re-growth appeared disorganized with a lower percentage of reconnections as compared with *Gigaspora* sp. Following healing, cytoplasmic/protoplasmic flow was re-established. Whatever the parameter (emission, contact and fusion of GHTs and number of GHTs and hyphal branches produced), no significant difference was highlighted between the MSR^acetone^ and MSR^control^ treatments (see data in supporting information), thus the impact of fungicides on the HHM was compared solely to the MSR^acetone^ control treatment.

### Impact of Fungicides on the Hyphal Healing Mechanism in *Gigaspora* sp. MUCL 52331

The impact of azoxystrobin, pencycuron, flutolanil and fenpropimorph at 0.02 and 2 mg L^–1^ was evaluated on the HHM in *Gigaspora* sp. MUCL 52331 (Tables 1, 2). A significant effect of the azoxystrobin^2 mgL^–1^^ treatment was noticed for GHTs emission (hr = 0.134, *P* < 0.001), contact (hr = 0.064, *P* < 0.001) and fusion (hr = 0.044, *P* < 0.001) (Table 2). All events were less often (or later in time) observed in presence of azoxystrobin^2 mgL^–1^^ as compared to the MSR^acetone^ treatment. The number of GHTs (*P* = 0.010) and hyphal branches (*P* = 0.004) was significantly lower in the azoxystrobin^2 mgL^–1^^ treatment as compared to the MSR^acetone^ treatment (Figure 2). Within a period of approximately 360 min following injury in the azoxystrobin^2 mgL^–1^^ treatment, the hyphae that did not produce GHTs showed retraction of cytoplasm in the opposite direction to the tip followed by formation of numerous septa inside the hyphae (result not shown). Although the percentage of all HHM observed events was lower in the azoxystrobin^0.2 mgL^–1^^ compared to the ones in the MSR^acetone^ treatment, no significant effect was revealed from the Cox Model (Table 2). The emission, contact and fusion of GHTs (Tables 1, 2) as well as number of GHTs and hyphal branches were not affected at both concentrations of pencycuron, flutolanil and fenpropimorph (Figure 2) as compared to the MSR^acetone^ treatment.

### Impact of Fungicides on Hyphal Healing Mechanism in *Rhizophagus irregularis* MUCL 41833

The impact of azoxystrobin, pencycuron, flutolanil and fenpropimorph at 0.02 and 2 mg L^–1^ was evaluated on the HHM of *R*. *irregularis* MUCL 41833 (Tables 1, 2). The results obtained from the Cox model revealed that the azoxystrobin^2 mgL^–1^^ treatment had a significant impact on GHTs contact and fusion as the hazard ratio’s were significantly less than 1 (hr = 0.317, *P* = 0.020, and hr = 0.294, *P* = 0.021, respectively) meaning that both events were less frequently observed within the azoxystrobin^2 mgL^–1^^ treatment as compared to the MSR^acetone^ one, while no significant effect of the azoxystrobin^2 mgL^–1^^ on GHTs emission was found. Although a decreased trend in the percentage of observed GHTs emission, contact, and fusion events was noted in the azoxystrobin^0.02 mgL^–1^^ treatment, no significant difference was reported by our analysis (Tables 1, 2). The number of GHTs and hyphal branches did not differ at both concentrations of azoxystrobin compared to the MSR^acetone^ treatment (Figure 3). After the formation of GHTs, the HHM process was inhibited in the azoxystrobin^2 mgL^–1^^ treatment. At the end of the evaluation, a limited growth of GHTs was observed and no septa was noticed in the injured hyphae of the azoxystrobin^2 mgL^–1^^ treatment with the exception of a septum in the area of the lesion.

The GHTs emission, contact, and fusion events were not significantly affected by both concentrations of pencycuron and flutolanil as compared to the MSR^acetone^ treatment (Tables 1, 2). In the case of flutolanil, the Cox proportional hazard model was not performed in the GHTs emission event because GHTs formation was observed in all the injuries (Table 1). Moreover, the number of GHTs and hyphal branches did not differ at both concentrations treatments (pencycuron and flutolanil) as compared to the MSR^acetone^ treatment (Figure 3).

Finally, the results of the Cox model showed that the fenpropimorph^2 mgL^–1^^ treatment reduced significantly the percentage of observed GHTs contact (hr = 0.274, *P* = 0.007) and fusion (hr = 0.294, *P* = 0.019) (Table 2). In presence of fenpropimorph^2 mgL^–1^^, the number of GHTs (*P* = 0.015) and the number of hyphal branches (*P* = 0.039) was significantly lower as compared to the MSR^acetone^ treatment (Figure 3). After the formation of GHTs, the HHM process was inhibited in the fenpropimorph^2 mgL^–1^^ treatment. At the end of the evaluation, a limited growth of GHTs was observed and no septa was noticed in the injured hyphae of fenpropimorph^2 mgL^–1^^ treatment with the exception of a septum in the area of the lesion. On the other hand, in the fenpropimorph^0.02 mgL^–1^^ treatment, GHTs emission (hr = 3.580, *P* < 0.001) and contact (hr = 2.238, *P* = 0.033) were stimulated significantly, showing this event more frequently than in the MSR^acetone^ treatment (Table 2).

## Discussion

Seed dressing or foliar application of chemical fungicides are considered essential agricultural practices to control fungal diseases in crops intended for market. They have different modes of action and can target a broad range or a specific group of fungi, but in numerous cases, they also affect non-target fungi. It is important to understand the effects of fungicides on these non-target organisms, because some of them (e.g., AM fungi) play key roles in plant growth and health and thus help to optimize strategies of fungicide applications. Here, the effects of fungicides with different modes of action were investigated on the HHM, a strategy allowing fungi to preserve their mycelial networks integrity, of two AM fungi (*Gigaspora* sp. MUCL 52331 and *R. irregularis* MUCL 41833) with contrasting life history strategies.

In the absence of fungicides, similar results to those obtained in the studies of de la Providencia et al. (2005) and de la Providencia et al. (2007) on *Glomeraceae* and *Gigasporaceae* grown *in vitro* were obtained, demonstrating that the approach considered in the present study was adequate for studying the HHM in presence of fungicides. These authors suggested that in *Gigasporaceae*, the HHM (i.e., GHTs contact and subsequent cytoplasmic/protoplasmic flow re-establishment) is the most probable way to maintain the viability of hyphae in adverse conditions. In contrast, in *Glomeraceae*, the HHM might increase the ability of the fungus to colonize the roots due to the proliferation of new branches at the apex of the injured hyphae as well as the reconnection of the affected area by networking several hyphae in a relatively small vicinity.

In presence of fungicides, differences were noticed between active ingredients (a.i.) and concentrations. Azoxystrobin was detrimental to both fungi at the concentration of 2 mg L^–1^ but not at 0.02 mg L^–1^. Pencycuron and flutolanil at both concentrations did not impact the HHM of the two fungi, while fenpropimorph had contrasting effects on both fungi. With *R. irregularis*, the HHM was stimulated at 0.02 mg L^–1^ and inhibited at 2 mg L^–1^ fenpropimorph, while no effect was observed at both concentrations in *Gigaspora* sp.

Azoxystrobin at 2 mg L^–1^ was the most threatening a.i. impacting all the HHM events in *Gigaspora* sp. and the contact and fusion of GHTs in *R*. *irregularis*, while at 0.02 mg L^–1^ no effects were noticed on any of the fungi. At the highest concentration of 2 mg L^–1^, an evident retraction of cytoplasm in the opposite direction to the tips and subsequent formation of numerous walls inside the hyphae was observed when GHTs were not produced [70%] or did not enter into contact [85%] in *Gigaspora* sp. In *R. irregularis*, the GHTs [87.5%] were produced but only a few contacts [37.5%] and fusions [31.2%] were observed and the hyphae that did not form GHTs presented a main septum in the lesion area and did not produce numerous walls as in *Gigaspora* sp. These results complement the observations made by Buysens et al. (2015) on the effect of azoxystrobin on AM fungi. These authors demonstrated under similar *in vitro* culture conditions that azoxystrobin at 0.1 mg L^–1^ (as formulation Amistar) affected spore production and hyphal length of *R*. *irregularis* MUCL 41833 associated with potato, while root colonization was reduced at 1 (a.i., or formulation Amistar) and 10 mg L^–1^ a.i. Greenhouse studies further demonstrated that soil drench with azoxystrobin inhibited root colonization of *Glomeraceae* members in sugarcane (Vuyyuru et al., 2018). Moreover, root colonization and enzymatic activity of *Funneliformis coronatum* associated with maize plants was also inhibited, demonstrating the fungicidal activity of this strobilurin, probably on the respiratory electron transfer within mitochondria, where succinate dehydrogenase is part of complex II (Diedhiou et al., 2004). Interestingly, foliar application of azoxystrobin did not impact AM fungal root colonization (Diedhiou et al., 2004; Hernández-Dorrego and Mestre-Parés, 2010; Campos et al., 2015). A similar trend was observed with kresoxim-methyl, another strobilurin fungicide (Diedhiou et al., 2004). Both strobilurin fungicides present low uptake into leaves and absence of phloem mobility (Bartlett et al., 2002) probably explaining the absence of effects on AM fungi in the roots. Interestingly, Buysens et al. (2015) evaluated that 0.75 mg L^–1^ azoxystrobin applied *in vitro* could be considered close to the recommended dosage of Amistar applied against *R*. *solani* in potato crop production (1500 g a.i., ha^–1^) (Service Public Fédéral, 2015). Therefore, the concentration of 2 mg L^–1^ of a.i., used at the place of hyphal injury was probably above the recommended field dosage, while 0.02 mg L^–1^ was below. From these results, it is speculative to assert that field recommended dosage may impact the fungus significantly, because several aspects (e.g., biodegradation, soil type and soil particles size) might affect fungicide exposure to AM fungi (O’Connor et al., 2002; Rivera-Becerril et al., 2017). However, our results complement those obtained by Buysens et al. (2015), suggesting that the direct application in the culture medium of azoxystrobin at concentrations equal or above 0.1 mg L^–1^ (a.i., or formulated product) affect the development of AM fungi, therefore not excluding an impact on its development under field conditions following soil application.

The strong negative impact of azoxystrobin at 2 mg L^–1^ on the HHM (in particular contact and fusion of GHTs) of both AM fungi could possibly been attributed to the perturbation of apical vesicular bodies in the hyphal tip called Spitzenkörper. This structure consists in many small vesicles, ribosomes and cytoskeletal elements present in GHTs (Steinberg, 2007) playing key roles in hyphal growth, orientation, branching and morphogenesis (Hickey et al., 2002; Harris et al., 2005). This structure has not formally been described in AM fungi. Although, in *Gigasporaceae*, a cluster of spherical lipid bodies was observed behind the apex of growing germ tubes (Bentivenga et al., 2013) as well as “spitzenkörper-like” structures at the hyphal apex of *Gigaspora margarita* (Ramos et al., 2008a,b). In *Glomeraceae* their presence has never been proved but only suggested (de la Providencia et al., 2005). A perturbation of these structures by chemicals could explain the lack of recognition between GHTs in *R. irregularis* and *Gigaspora* sp. The effects of chemical molecules with fungicidal effects on the Spitzenkörper remains poorly investigated. Carbendazim, an inhibitor of ß-tubulin assembly in mitosis, was reported to inhibit completely the Spitzenkörper on living hyphal tip cells in *Fusarium acuminatum* (Ascomycota) (Howard and Aist, 1977, 1980). Instead, in *Sclerotium rolfsii* (Basidiomycota), the Spitzenkörper position was displaced within the apical hyphal zone changing the growth direction when hyphae were treated with cyproconazole, an inhibitor of ergosterol synthesis, causing alteration of the microtubule cytoskeleton and abnormal wall deposition (Roberson et al., 1989). These studies demonstrate that chemicals may impact growth and orientation of hyphae, hence affecting other processes such as anastomosis between branches of intact hyphae or contact and fusion of GHTs in injured hyphae. Although this has to be demonstrated for those fungi and extended to AM fungi with molecules such as azoxystrobin.

Pencycuron at 0.02 and 2 mg L^–1^ did not impact the HHM of both AM fungal strains. Buysens et al. (2015) also demonstrated that pencycuron at 0.01 mg L^–1^ a.i., did not affect the ERM development or root colonization. To the contrary, at the concentration of 0.5 mg L^–1^ a.i., or formulation and 5 mg L^–1^ a.i., root colonization of *R. irregularis* was reduced. One, 10, 100 mg L^–1^ a.i., or formulation further impacted spore germination (Buysens et al., 2015). These authors calculated that a concentration of 0.25 mg L^–1^ used *in vitro* was equivalent to the recommended dose of Monceren applied in the field to control *R*. *solani* in potato crop production (500 g ha^–1^ a.i.). Our results suggested that the direct application of pencycuron at 0.02 and 2 mg L^–1^ had no noticeable effects on the HHM, while higher concentrations above the recommended dose calculated *in vitro* may affect other processes (e.g., spore germination, root colonization) of AM fungal life cycle. Pencycuron is the only fungicide of the phenylurea group, which also includes herbicides for weed control in agricultural and non-agricultural systems (Navarro et al., 2012) and is known to inhibit cell division of *Rhizoctonia* sp. The absence of effect of pencycuron on the HHM at concentrations up to 2 mg L^–1^ may suggest that the enzymes involved in cell division in AM fungi are less sensitive than those in *Rhizoctonia* sp. A study under greenhouse conditions nonetheless demonstrated that the cytokinin-like growth regulator thidiazuron (phenylurea compounds) applied on leaves at 20 mg L^–1^ (91 μM) decreased root colonization of *R*. *intraradices* associated with the perennial grass *Miscanthus giganteus* (Schmidt et al., 2017), suggesting an indirect effect on AM colonization by a decrease in active cytokinines in the plant shoot. A modification in plant metabolism by the application of pencycuron on the leaves may thus impact AM fungal colonization more severely than a direct contact with the molecule.

Flutolanil at both concentrations did not affect the HHM of *Gigaspora* sp. and *R*. *irregularis*. This corroborates partially the study of Buysens et al. (2015). These authors noticed that flutolanil at 0.1 mg L^–1^ a.i., did not affect ERM development, while spore germination was reduced at 10 and 100 mg L^–1^ a.i., or formulation Monarch and root colonization decreased at 1 mg L^–1^ a.i., or formulation Monarch and 10 mg L^–1^ a.i. They suggested that flutolanil was more detrimental to the intraradical structures (hyphae and arbuscules) than ERM structures because it is an inhibitor of the succinate dehydrogenase and has a high systemic activity. Systemic fungicides are thought to be more detrimental to AM fungi because of their accumulation in or on the roots (Diedhiou et al., 2004; Burrows and Ahmed, 2007; Jin et al., 2013). The absence of impact of flutolanil at 0.02 and 2 mg L^–1^ on the HHM of AM fungi suggests that the enzymatic metabolisms of the respiration pathway in AM fungi are probably less sensitive to flutolanil than those of Basidiomycota plant pathogens. In addition, 0.02 and 2 mg L^–1^ concentrations could be considered lower or higher, respectively, than the recommended dose reported by Buysens et al. (2015). These authors hypothesized a value of 0.09 mg L^–1^ *in vitro* considering the recommended dose of Monarch applied to control *R*. *solani* in potato crop production (184 g ha^–1^ a.i.). Our results thus suggest a tolerance of AM fungi to the direct application of flutolanil even at a concentration above the recommended dose estimated *in vitro*.

Fenpropimorph at the highest concentration impacted the HHM in *R. irregularis*, while the opposite was noticed at the lowest concentration. Curiously, no effect was observed on *Gigaspora* sp. It is not excluded that these differences may be related to the sterol composition of both fungi. Indeed, a variation in the composition and content of sterols has been observed in the spores of AM fungal species in the *Glomeraceae*, *Gigasporaceae*, and *Acaulosporaceae* families (Grandmougin-Ferjani et al., 1999), not excluding the possibility of differences in the GHTs.

Sterols accumulation at the fungal growing tips supports hyphal growth facilitating apical endocytosis and organizing the cytoskeleton (Steinberg, 2007). Differences in sterol composition in GHTs may lead to differences in sensitivity to SBIs fungicides as fenpropimorph. In *R. irregularis*, GHT production was not affected by the high concentration of fenpropimorph, but reduced contacts and thus fusions were observed, suggesting perturbation of the GHTs recognition as with azoxystrobin. A common attraction (positive tropism) between GHTs was observed with *Gigasporaceae* members and suggested in *Glomeraceae* (de la Providencia et al., 2005). These authors hypothesized that the growth of both GHTs toward each other was due to elicitation of diffusible substance and that this mechanism was led by one GHT in a sequence of a signaling-response.

The stimulatory effect observed on HHM of *R*. *irregularis* at low concentration (0.02 mg L^–1^) and opposite observed at high concentration (2 mg L^–1^) of fenpropimorph could be related to a phenomenon named hormesis. The hormetic effect is a biphasic dose response characterized by temporal stimulation at low doses and inhibition at high doses of a stressor agent, suggesting an adaptive response that can be either directly induced or be the result of overcompensation after the alteration of homeostasis in a biological process (Calabrese and Baldwin, 2002). The hormetic effect of fungicides on mycelial growth and virulence have been previously reported in Oomycota, Ascomycota and Basidiomycota plant pathogens (Garzon and Flores, 2013; Lu et al., 2018; Zhang R. et al., 2019). To the best of our knowledge, hormesis has never been addressed in Glomeromycota and would deserve attention owing to the importance of these organisms for crops (Hage-Ahmed et al., 2019).

A strong impact of fenpropimorph at concentrations equal or above 0.2 mg L^–1^ under *in vitro* culture conditions was observed by Campagnac et al. (2008) and Zocco et al. (2008) on ERM development of *R. irregularis* (hyphal growth, spore production and mycelium architecture). This impact resulted in a decreased capacity of the fungus to transport P to the host plant and a drastic decrease of alkaline phosphatase and succinate dehydrogenase activities measured in the ERM (Zocco et al., 2011). With the same AM fungus, an induction of oxidative stress was observed in presence of fenpropimorph at 10 mg L^–1^, suggesting the perturbation of other biological processes such as response to biotic and abiotic environmental stimuli (González-Guerrero et al., 2010).

In conclusion, this work reports for the first time the effects of fungicides with different modes of action on the HHM in two phylogenically distant AM fungi (*R. irregularis* MUCL 41833 and *Gigaspora* sp. MUCL 52331). Azoxystrobin (a broad-spectrum fungicide) was the more detrimental on both fungi at concentration slightly above the recommended field dosage. Conversely, the contact fungicide pencycuron (*Rhizoctonia*-specific) and flutolanil (Basidiomycota-specific) did not impact any of the two AM fungi, while fenpropimorph only impacted *R. irregularis* (stimulating at low and inhibiting at high concentrations). The mechanisms behind the observed results remains to be elucidated, but perturbation in the still-to-be firmly demonstrated spitzenkörper or sterols content as well as a process of hormesis are avenues to be explored. This study broadens our knowledge on the impact of fungicides on AM fungi and opens new avenues for the rationale management of chemical inputs to control pathogenic fungi while limiting their impact on AM fungi. It also increases our knowledge of the different strategies for AM fungal colony to survive under adverse conditions in agricultural soils.

## Data Availability Statement

The original contributions presented in the study are included in the article/Supplementary Material, further inquiries can be directed to the corresponding author/s.

## Author Contributions

VHR-M: data collection, analysis and interpretation, drafting the work, commentaries corrections, final approval, and agreement with all aspects of the work. MC-S: data collection, analysis and interpretation, draft work commentaries and corrections, final approval, and agreement with all aspects of the work. VB: analysis and interpretation of the data, draft correction and final approval and agreement with all aspects of the work. MG-R: contribution to the development of the experiment, draft correction and final approval, and agreement with all aspects of the work. SD: Substantial contributions to the conception and design of the experiments, interpretation of the data, draft corrections final approval, and agreement with all aspects of the work. All authors contributed to the article and approved the submitted version.

## Conflict of Interest

The authors declare that the research was conducted in the absence of any commercial or financial relationships that could be construed as a potential conflict of interest.

## Footnotes

1 http://www.mycorrhiza.be/ginco-bel
