# Supplementary material for: Fungicides With Contrasting Mode of Action Differentially Affect Hyphal Healing Mechanism in Gigaspora sp. and Rhizophagus irregularis
Source: Front Plant Sci. 2021 Mar 10;12:642094. doi: 10.3389/fpls.2021.642094 (PMC7989550; doi:10.3389/fpls.2021.642094)
Supplement: Supplementary file 1 [file Data_Sheet_1.docx]

**Table S1.** Descriptive data percentages of hyphal branches production, growing hyphal tips (GHT) emission, contact and fusion of the hyphal healing mechanism (HHM) of *Gigaspora sp.* MUCL 52331 and *Rhizophagus irregularis* MUCL 41833 in the control treatment (MSR medium without acetone and fungicides) and acetone control treatment (without fungicides).

| ***Gigaspora* sp. MUCL 52331** | | | ***Rhizophagus irregularis* MUCL 41833** | |
| --- | --- | --- | --- | --- |
|  | **Control Treatment** | **Control Acetone** | **Control Treatment** | **Control Acetone** |
| **Azoxystrobin (group)** |  |  |  |  |
| Hyphal branches (%) | 100 | 94.4 | 93.3 | 73.7 |
| GHT emission (%) | 100 | 94.4 | 100 | 100 |
| GHT contact (%) | 89.5 | 88.9 | 80 | 73.7 |
| GHT fusion (%) | 89.5 | 88.9 | 66.7 | 68.4 |
| Number of injured hyphae (n) | 19 | 18 | 15 | 19 |
| **Pencycuron (group)** |  |  |  |  |
| Hyphal branches (%) | 88.2 | 81.2 | 88.9 | 94.4 |
| GHT emission (%) | 100 | 87.5 | 94.4 | 94.7 |
| GHT contact (%) | 86.6 | 81.2 | 83.3 | 89.5 |
| GHT fusion (%) | 86.6 | 81.2 | 77.7 | 73.7 |
| Number of injured hyphae (n) | 15 | 16 | 18 | 19 |
| **Flutolanil (group)** |  |  |  |  |
| Hyphal branches (%) | 75 | 72.2 | 100 | 94.7 |
| GHT emission (%) | 100 | 94.4 | 100 | 100 |
| GHT contact (%) | 81.2 | 83.3 | 82.3 | 89.5 |
| GHT fusion (%) | 81.2 | 83.3 | 76.5 | 73.7 |
| Number of injured hyphae (n) | 16 | 18 | 17 | 19 |
| **Fenpropimorph (group)** |  |  |  |  |
| Hyphal branches (%) | 78.6 | 87.5 | 94.1 | 85 |
| GHT emission (%) | 100 | 100 | 100 | 90 |
| GHT contact (%) | 92.8 | 100 | 76.5 | 85 |
| GHT fusion (%) | 92.8 | 87.5 | 70.6 | 70 |
| Number of injured hyphae (n) | 14 | 16 | 17 | 19 |

**Table S2.** Number of growing hyphal tips (GHT) and their hyphal branches produced in the hyphal healing mechanism (HHM) of *Gigaspora* sp. MUCL 52331 and *Rhizophagus irregularis* MUCL 41833 in the control treatment (MSR medium without acetone and fungicides) and acetone control treatment (without fungicides). Data were obtained 48h after hyphal physical injury and addition of MSR medium containing or not acetone at the place of injury.

| ***Gigaspora* sp. MUCL 52331** | | | ***Rhizophagus irregularis* MUCL 41833** | |
| --- | --- | --- | --- | --- |
|  | **Control Treatment** | **Control Acetone** | **Control Treatment** | **Control Acetone** |
| **Azoxystrobin (group)** |  |  |  |  |
| Number of GHTs | 2.6 ± 0.3 | 2.2 ± 0.2 | 4.5 ± 0.5 | 4.2 ± 0.4 |
| Number of hyphal branches | 4.7 ± 0.8 | 3.3 ± 0.9 | 5.2 ± 1.1 | 5.5 ± 1.8 |
| Number of injured hyphae (n) | 19 | 18 | 15 | 19 |
| **Pencycuron (group)** |  |  |  |  |
| Number of GHTs | 3.3 ± 0.3 | 3.1 ± 0.4 | 3.9 ± 0.4 | 4.3 ± 1.9 |
| Number of hyphal branches | 6.5 ± 1.4 | 4.9 ± 0.8 | 5.4 ± 0.4 | 4.3 ± 1.1 |
| Number of injured hyphae (n) | 15 | 16 | 18 | 19 |
| **Flutolanil (group)** |  |  |  |  |
| Number of GHTs | 2.7 ± 0.3 | 3.3 ± 0.3 | 4.6 ± 0.4 | 5.0 ± 0.3 |
| Number of hyphal branches | 4.3 ± 1.6 | 4.5 ± 1.0 | 3.1 ± 0.9 | 3.1 ± 0.7 |
| Number of injured hyphae (n) | 16 | 18 | 17 | 19 |
| **Fenpropimorph (group)** |  |  |  |  |
| Number of GHTs | 3.1 ± 0.2 | 2.7 ± 0.2 | 3.3 ± 0.3 | 4.0 ± 0.4 |
| Number of hyphal branches | 4.9 ± 1.6 | 7.5 ± 1.7 | 5.1 ± 1.7 | 7.3 ± 1.7 |
| Number of injured hyphae (n) | 14 | 16 | 17 | 19 |

**Table S3.** Results of the Cox proportional hazards regression analysis on growing hyphal tips (GHT) emission, contact and fusion events of the hyphal healing mechanism (HHM) for *Gigaspora* sp. MUCL 52331 and *Rhizophagus irregularis* MUCL 41833 in the control treatment (MSR medium without acetone and fungicides).

|  | *Gigaspora* sp. MUCL 52331 | | | *Rhizophagus irregularis* MUCL 41833 | | |
| --- | --- | --- | --- | --- | --- | --- |
|  | **β** | **Hazard ratio** | **P-Value** | **β** | **Hazard ratio** | **P-Value** |
| **Azoxystrobin (group)** |  |  |  |  |  |  |
| GHT emission | -0.062 | 0.940 | 0.858 | 0.640 | 1.896 | 0.085 |
|  |  |  |  |  |  |  |
| GHT contact | -0.147 | 0.863 | 0.675 | 0.181 | 1.198 | 0.653 |
|  |  |  |  |  |  |  |
| GHT fusion | -0.120 | 0.886 | 0.731 | -0.178 | 0.837 | 0.677 |
| **Pencycuron**  **(group)** |  |  |  |  |  |  |
| GHT emission | 0.922 | 2.515 | 0.106 | -0.114 | 0.892 | 0.739 |
|  |  |  |  |  |  |  |
| GHT contact | 0.305 | 1.356 | 0.444 | -0.099 | 0.906 | 0.781 |
|  |  |  |  |  |  |  |
| GHT fusion | 0.067 | 1.070 | 0.864 | 0.198 | 1.219 | 0.603 |
| **Flutolanil**  **(group)** |  |  |  |  |  |  |
| GHT emission | -0.028 | 0.972 | 0.936 | NA | NA | NA |
|  |  |  |  |  |  |  |
| GHT contact | 0.168 | 1.182 | 0.659 | -0.269 | 0.764 | 0.468 |
|  |  |  |  |  |  |  |
| GHT fusion | -0.076 | 0.927 | 0.842 | 0.301 | 1.351 | 0.437 |
| **Fenpropimorph**  **(group)** |  |  |  |  |  |  |
| GHT emission | 0.155 | 1.168 | 0.684 | 0.651 | 1.917 | 0.070 |
|  |  |  |  |  |  |  |
| GHT contact | -0.005 | 0.995 | 0.989 | 0.074 | 1.077 | 0.843 |
|  |  |  |  |  |  |  |
| GHT fusion | 0.528 | 1.696 | 0.179 | 0.287 | 1.333 | 0.467 |

Values were considered significant at P < 0.05 compared to the acetone control treatment.

β = Regression coefficient.

Hazard ratio = Exp (β).

NA = Not applicable.
